# On the Free Vibrations of Non-Classically Damped Locally Resonant Metamaterial Plates

**DOI:** 10.3390/nano12030541

**Published:** 2022-02-05

**Authors:** Andrea Francesco Russillo, Giuseppe Failla, Ada Amendola, Raimondo Luciano

**Affiliations:** 1 Department of Civil, Energy, Environmental and Materials Engineering (DICEAM), University of Reggio Calabria, Via Graziella, 89124 Reggio Calabria, Italy; andreaf.russillo@unirc.it; 2 Department of Civil Engineering, University of Salerno, 84084 Fisciano, Italy; adaamendola1@unisa.it; 3 Department of Civil Engineering, Parthenope University of Naples, 80133 Naples, Italy; raimondo.luciano@uniparthenope.it

**Keywords:** metamaterial plate, local resonance, dynamic-stiffness model, contour-integral algorithm

## Abstract

In this paper, the focus is on the free vibrations of locally resonant metamaterial plates with viscously damped resonators. Upon formulating a dynamic-stiffness model where the resonators are represented via pertinent reaction forces depending on the deflections of the attachment points, the complex eigenvalues are calculated by a contour-integral algorithm introduced in the literature for general nonlinear eigenvalue problems. The interest in the proposed approach is twofold. The dynamic-stiffness model involves a limited number of generalised coordinates compared to the nodal degrees of freedom of a standard finite-element model, and the contour-integral algorithm proves successful in evaluating all complex eigenvalues, without missing any one, with remarkable computational efficiency. Numerical results are presented for Lévy plates, but are readily extendible to other plate theories. Finally, an ad hoc dynamic-stiffness approach is formulated to calculate the frequency response of the plate under arbitrarily placed loads, which is of particular interest to investigate its elastic wave attenuation properties.

## 1. Introduction

There is a considerable body of recent literature on locally resonant metamaterial plates (LRMPs), i.e., plates engineered with a periodic array of small resonators. Indeed, these plates exhibit the inherent attenuation properties of elastic waves that make them ideally suitable for several applications in dynamics. Typically, the resonators may be distributed over the external surface of homogeneous or composite plates [1,2,3,4,5,6,7,8,9,10] or, alternatively, they may be embedded within the core matrix of sandwich plates [11,12,13].

Computational models of LRMPs generally rely on standard finite-element (FE) analysis. The model involves a standard FE for the plate, which is coupled with concentrated mass–spring or mass–spring–damper subsystems representing the resonators [6,8]. In some cases, the resonators are modelled by FEs as well [12,13].

A very appealing approach for modelling bare plates, i.e., plates without resonators, is a dynamic-stiffness approach, as it ensures a very accurate description of the plate dynamics with a limited number of generalised coordinates compared to a standard FE model. For instance, in a comprehensive treatment for plates of various types, Banerjee and coworkers [14,15,16,17,18,19,20,21] developed dynamic-stiffness models where a few coefficients of appropriate Fourier series expansions proved capable of representing, with remarkable accuracy, the plate dynamic response. In this context, natural frequencies of the undamped modes were calculated by the powerful Wittrick–Williams algorithm [17,22], without missing any one and including multiple ones. As for LRMPs, a pertinent dynamic-stiffness model and an ad hoc formulation of the Wittrick–Williams algorithm were recently proposed by Russillo et al. [23].

A main limitation in the existing dynamic-stiffness models for LRMPs is that no damping is considered [17,22]. Yet, damping is often intentionally introduced into LRMPs, for instance by endowing the resonators with viscous dampers [24]. However, dealing with the free vibrations of dynamic-stiffness models for damped LRMPs poses severe computational challenges, because the eigenvalues are complex and the Wittrick–Williams algorithm, i.e., the classical algorithm used in dynamic-stiffness approaches, no longer applies. Moreover, a further difficulty is that the complex eigenvalues are very close to each other, as a result of local resonance. In view of these complications, no existing study deals with the free vibrations of dynamic-stiffness models for damped LRMPs, to the best of the authors’ knowledge.

This paper addresses the free vibrations of LRMPs with viscously damped resonators, focusing on Lévy plates. Two main novelties are introduced. A dynamic-stiffness formulation is adopted, which builds on the formulation proposed by Banerjee and coworkers for bare plates [14,15,16,17,18,19,20,21] and considers the resonators via pertinent reaction forces, expressed in terms of the deflections of the attachment points by a frequency-dependent stiffness obtained from the resonator motion equations. In this way, the size of the dynamic-stiffness model depends only on the number of generalised coordinates of the plate and does not include any degrees of freedom of the resonators, with a significant computational advantage as, in general, LRMPs involve a very large number of resonators. Next, the free-vibration problem is tackled by a contour-integral algorithm [25,26,27,28], introduced a decade ago for nonlinear eigenvalue problems and used by the authors, very recently, for the free-vibration problem of damped locally resonant sandwich beams [29]. Comparing the proposed dynamic-stiffness approach to a standard FE one implemented in Abaqus demonstrates that: (1) the size of the dynamic-stiffness model is much smaller than the size of the FE model required to attain the same accuracy; (2) the contour-integral algorithm is capable of calculating accurately all the complex eigenvalues, without missing any one; (3) calculating the eigenvalues by the dynamic-stiffness approach via the contour-integral algorithm is computationally more efficient than by the FE method via standard eigensolvers. The formulation presented here for Lévy plates is readily extendible to Kirchhoff ones with arbitrary boundary conditions (BCs). Finally, the paper proposes a dynamic-stiffness approach to calculate the frequency response of the LRMP under arbitrarily placed concentrated loads, for the purpose of investigating the elastic wave attenuation properties of the plate.

The paper is organised as follows. The dynamic-stiffness model of Lévy LRMPs is described in Section 2. Details on the contour-integral algorithm are illustrated in Section 3, and the dynamic-stiffness approach to calculate the frequency response is described in Section 4. A numerical example is discussed in Section 5.

## 2. Dynamic-Stiffness Model

**Figure 1:**
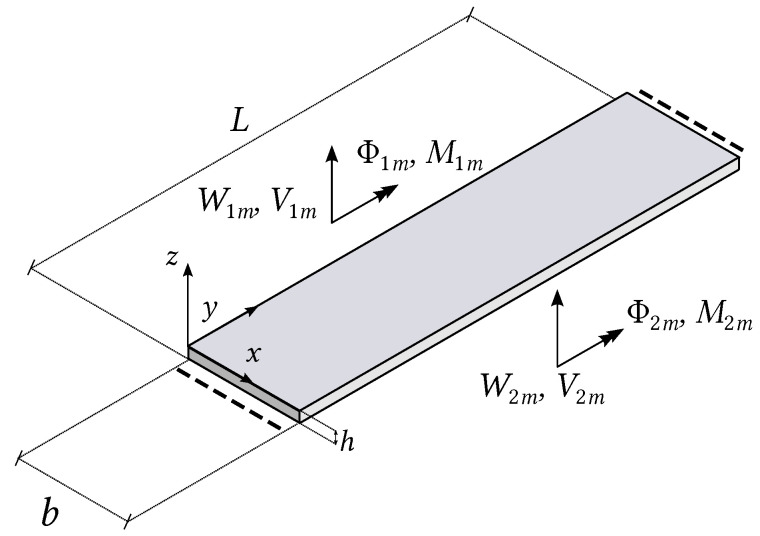
Generalised displacements and forces for a single plate strip with simply supported edges parallel with the *x*-axis.

Consider the equation of motion of a Kirchhoff plate strip in Figure 1 where the rotational inertia is taken into account [14]:

(1) $$D\left(\frac{\partial^{4}w}{\partial x^{4}}+2\frac{\partial^{4}w}{\partial x^{2}\partial y^{2}}+\frac{\partial^{4}w}{\partial y^{4}}\right)+\rho h\frac{\partial^{2}w}{\partial t^{2}}-\frac{1}{12}\rho h^{3}\left(\frac{\partial^{4}w}{\partial x^{2}\partial t^{2}}+\frac{\partial^{4}w}{\partial y^{2}\partial t^{2}}\right)=0$$

w(x,y,t) being the transverse deflection, *h* the thickness, ρ the volumetric mass density, and *D* the bending rigidity of the plate. A solution that enforces simply supported BCs at y=0 and y=L is considered:

(2) $$w\left(x,y,t\right)=\sum_{m=1}^{\infty }W_{m}\left(x\right)\sin\left(\alpha_{m}y\right)e^{i\omega t}$$

where $\alpha_{m}=\frac{m\pi }{L}$ for m=1,2,…,∞. Replacing Equation (2) in Equation (1) yields:

(3) $$\frac{d^{4}W_{m}}{dx^{4}}+\left(\frac{\rho h^{3}\omega^{2}}{12D}-2\alpha_{m}^{2}\right)\frac{d^{2}W_{m}}{dx^{2}}+\left(\alpha_{m}^{4}-\frac{\rho h\omega^{2}}{D}-\frac{\rho h^{3}\omega^{2}}{12D}\alpha_{m}^{2}\right)W_{m}=0$$

Introduce the vectors collecting the generalised displacements and forces along the two unconstrained edges of the strip:

(4) $$u_{m}^{s}=\left[\begin{array}{c} W_{1m}\\ \Phi_{1m}\\ W_{2m}\\ \Phi_{2m} \end{array}\right]=\left[\begin{array}{c} W_{m}\left(0\right)\\ \Phi_{ym}\left(0\right)\\ W_{m}\left(b\right)\\ \Phi_{ym}\left(b\right) \end{array}\right];\;f_{m}^{s}=\left[\begin{array}{c} V_{1m}\\ M_{1m}\\ V_{2m}\\ M_{2m} \end{array}\right]=\left[\begin{array}{c} -V_{xm}\left(0\right)\\ -M_{xm}\left(0\right)\\ V_{xm}\left(b\right)\\ M_{xm}\left(b\right) \end{array}\right]$$

where $\Phi_{ym}\left(x\right)=-dW_{m}/dx$ is the rotation in the xz plane and $V_{xm}\left(x\right)$ and $M_{xm}\left(x\right)$ are the shear force and bending moment per unit length along the edge of the strip. Making use of the Kirchhoff plate equations providing $V_{xm}\left(x\right)$ and $M_{xm}$ as functions of $W_{m}\left(x\right)$, the following matrix relation is obtained [14]:

(5) $$f_{m}^{s}=D_{m}^{s}\left(\omega \right)u_{m}^{s}$$

where $D_{m}^{s}$ is the dynamic-stiffness matrix of the single strip for m=1,2,…,∞.

The dynamic-stiffness matrix of a plate consisting of $n_{e}-1$ strips ($n_{e}$ is number of lines) is assembled in a finite-element fashion, and the following equation of equilibrium holds:

(6) $$f_{m}=D_{m}\left(\omega \right)u_{m}$$

for m=1,2,…,∞, where $f_{m}={\left[\begin{array}{ccc} f_{m,1}^{T} & \ldots & f_{m,n_{e}}^{T} \end{array}\right]}^{T}$ and $u_{m}={\left[\begin{array}{ccc} u_{m,1}^{T} & \ldots & u_{m,n_{e}}^{T} \end{array}\right]}^{T}$. Truncating the Fourier series (2) up to *N* terms, Equation (6) can be equivalently written in matrix form:

(7) $$\underset{f}{\underset{︸}{\left[\begin{array}{c} f_{1}\\ \vdots \\ f_{N} \end{array}\right]}}=\underset{D(\omega )}{\underset{︸}{\left[\begin{array}{ccc} D_{1}\left(\omega \right) & \ldots & 0\\ \vdots & \ddots & \vdots \\ 0 & \ldots & D_{N}\left(\omega \right) \end{array}\right]}}\underset{u}{\underset{︸}{\left[\begin{array}{c} u_{1}\\ \vdots \\ u_{N} \end{array}\right]}}$$

It is noticed that the displacement/force $q_{i}\left(y\right)$ along the ith line, either at the boundary or between two adjacent strips of the assembled plate, can be expanded into a sine Fourier series:

(8) $$q_{i}\left(y\right)=\sum_{m=1}^{\infty }Q_{im}\sin\left(\alpha_{m}y\right)$$

where the coefficients $Q_{im}$ are given as:

(9) $$Q_{im}=\frac{2}{L}\int_{0}^{L}q_{i}\left(y\right)\sin\left(\alpha_{m}y\right)\;dy$$

Now, consider a resonator placed on the *i*th line at $y=y_{j}$. Resonators attached along the *i*th line exert a force per unit length:

(10) $$f_{i}\left(y\right)=-k_{eq}\left(\omega \right)\sum_{j=1}^{n_{s}}w_{i}\left(y_{j}\right)\delta \left(y-y_{j}\right)$$

where δ is Dirac’s delta function, $n_{s}$ is the number of resonators attached along the *i*th line, and $k_{eq}\left(\omega \right)$ is the frequency-dependent stiffness of the resonator, which can be obtained from its dynamic-stiffness matrix, as demonstrated by the authors in [23,29,30,31].

By means of Equation (8), the displacement of the attachment point is:

(11) $$w_{i}\left(y_{j}\right)=\sum_{\ell =1}^{\infty }W_{i\ell }\sin\left(\alpha_{\ell }y_{j}\right)$$

Replacing Equation (11) in Equation (10) yields:

(12) $$f_{i}\left(y\right)=-k_{eq}\left(\omega \right)\sum_{j=1}^{n_{s}}\sum_{\ell =1}^{\infty }W_{i\ell }\sin\left(\alpha_{\ell }y_{j}\right)\delta \left(y-y_{j}\right)$$

Applying the transformation in Equation (9) to Equation (12) gives the Fourier series coefficients associated with the reaction forces exerted by the resonators:

(13) $$F_{im}=-\frac{2k_{eq}\left(\omega \right)}{L}\sum_{j=1}^{n_{s}}\sum_{\ell =1}^{\infty }W_{i\ell }\sin\left(\alpha_{\ell }y_{j}\right)\sin\left(\alpha_{m}y_{j}\right)$$

Equation (13) is readily written in matrix form as:

(14) $$\left[\begin{array}{c} F_{i1}\\ F_{i2}\\ \vdots \end{array}\right]=-D_{\mathrm{res}}\left(\omega \right)\left[\begin{array}{c} W_{i1}\\ W_{i2}\\ \vdots \end{array}\right]$$

matrix $D_{\mathrm{res}}$ being defined as follows:

(15) $${\left(D_{\mathrm{res}}\right)}_{m\ell }\left(\omega \right)=\frac{2k_{eq}\left(\omega \right)}{L}\sum_{j=1}^{n_{s}}\sin\left(\alpha_{m}y_{j}\right)\sin\left(\alpha_{\ell }y_{j}\right)$$

Matrix $D_{\mathrm{res}}$ in Equation (15) is the dynamic-stiffness matrix associated with the resonators. Now, consider Equation (15), and define the following diagonal matrix with $n_{e}$ submatrices:

(16) $${\bar{D}}_{m\ell }\left(\omega \right)=\left[\begin{array}{ccc} \Gamma_{m\ell }\left(\omega \right) & \ldots & 0\\ \vdots & \ddots & \vdots \\ 0 & \ldots & \Gamma_{m\ell }\left(\omega \right) \end{array}\right]\;\Gamma_{m\ell }\left(\omega \right)=\left[\begin{array}{cc} {\left(D_{\mathrm{res}}\right)}_{m\ell }\left(\omega \right) & 0\\ 0 & 0 \end{array}\right]$$

Notice that the *i*th block refers to the *i*th line of the assembled plate. Using Equation (16) and truncating the Fourier series (13) up to *N* terms, the following relation is readily obtained, which mirrors Equation (7):

(17) $$\underset{f}{\underset{︸}{\left[\begin{array}{c} f_{1}\\ f_{2}\\ \vdots \\ f_{N} \end{array}\right]}}=\underset{D_{r}\left(\omega \right)}{\underset{︸}{\left[\begin{array}{cccc} {\bar{D}}_{11}\left(\omega \right) & {\bar{D}}_{12}\left(\omega \right) & \ldots & {\bar{D}}_{1N}\\ {\bar{D}}_{21}\left(\omega \right) & {\bar{D}}_{22}\left(\omega \right) & \ldots & {\bar{D}}_{2N}\\ \vdots & \vdots & \ddots & \vdots \\ {\bar{D}}_{N1}\left(\omega \right) & {\bar{D}}_{N2}\left(\omega \right) & \ldots & {\bar{D}}_{NN} \end{array}\right]}}\underset{u}{\underset{︸}{\left[\begin{array}{c} u_{1}\\ u_{2}\\ \vdots \\ u_{N} \end{array}\right]}}$$

Finally, replacing Equation (17) for f in Equation (7) leads to the following nonlinear eigenproblem for the free-vibration response:

(18) $$\tilde{D}\left(\omega \right)u=\left(D\left(\omega \right)+D_{r}\left(\omega \right)\right)u=0$$

The matrix $\tilde{D}\left(\omega \right)$ is the dynamic-stiffness matrix of the Lévy LRMP.

## 3. Contour-Integral Algorithm

In order to compute the eigenvalues of the Lévy LRMP, it is necessary to solve the nonlinear eigenproblem given by Equation (18). The presence of viscous dampers within the resonators makes the system non-classically damped and, consequently, the eigenvalues are complex. In this case, the usual algorithm employed to solve the eigenproblems associated with dynamic-stiffness models, that is the Wittrick–Williams algorithm, is no longer applicable. Therefore, the recently introduced contour-integral algorithm formulated by Asakura and coworkers [26] is used here, for the first time, to solve this challenging problem. The application of the algorithm follows these steps:

1. Select a circle on the complex plane $\Gamma =\gamma_{0}+\rho_{0}e^{i\theta }$ with centre $\gamma_{0}$ and radius $\rho_{0}$ with 0≤θ≤2π;
2. Compute two complex random source matrices U and V having dimensions $n_{0}\times L_{0}$, $n_{0}$ being the size of the dynamic-stiffness matrix $\tilde{D}\left(\omega \right)$ in Equation (18) and $L_{0}$ the number of source vectors collected in U and V;
3. Compute the shifted and scaled moments $M_{k}$ using the $N_{0}$-point trapezoidal rule:
  $$\begin{array}{c} S_{k}=\frac{1}{N_{0}}\sum_{j=0}^{N_{0}-1}{\left(\frac{\omega_{j}-\gamma_{0}}{\rho_{0}}\right)}^{k+1}\tilde{D}{\left(\omega_{j}\right)}^{-1}V,\;k=0,1,\ldots ,2K-1\\ M_{k}=U^{H}S_{k} \end{array}$$
  with *K* the maximum moment degree considered for the moment and $U^{H}$ the Hermitian transpose of U;
4. Construct the Hankel matrices ${\hat{H}}_{KL_{0}}$ and ${\hat{H}}_{KL_{0}}^{<}\in C^{KL_{0}\times KL_{0}}$ such that:
  $${\hat{H}}_{KL_{0}}={\left[M_{i+j-2}\right]}_{i,j=1}^{K}\;{\hat{H}}_{KL_{0}}^{<}={\left[M_{i+j-1}\right]}_{i,j=1}^{K};$$
5. Perform the singular-value decomposition of ${\hat{H}}_{KL_{0}}$;
6. Omit small singular-value components $\sigma_{i}<\epsilon \cdot \max_{i}\sigma_{i}$; set the number $\tilde{m}$ of remaining singular value components $\left(\tilde{m}<KL_{0}\right)$; construct ${\hat{H}}_{\tilde{m}}$ and ${\hat{H}}_{\tilde{m}}^{<}$ extracting the principal submatrix with maximum index $\tilde{m}$ from ${\hat{H}}_{KL_{0}}$ and ${\hat{H}}_{KL_{0}}^{<}$, i.e.,
  $${\hat{H}}_{\tilde{m}}={\hat{H}}_{KL_{0}}\left(1:\tilde{m},1:\tilde{m}\right);\;{\hat{H}}_{\tilde{m}}^{<}={\hat{H}}_{KL_{0}}^{<}\left(1:\tilde{m},1:\tilde{m}\right);$$
7. Compute the eigenvalues $\zeta_{j}$ of the linear pencil:
  $${\hat{H}}_{\tilde{m}}^{<}=\zeta {\hat{H}}_{\tilde{m}};$$
8. Calculate the eigenvalues:
  $$\omega_{j}=\gamma_{0}+\rho_{0}\zeta_{j},\;j=1,\ldots ,\tilde{m}.$$

The algorithm allows calculating all the eigenvalues $\omega_{j}$ of the nonlinear eigenproblem (18) that fall within the selected circle Γ, including multiple roots [25,26,27].

## 4. Frequency Response

The frequency response of a Lévy LRMP can be computed by considering the equilibrium equation:

(19) $$f=\tilde{D}\left(\omega \right)u$$

A distributed force $p_{i}\left(y\right)$ and moment $m_{i}\left(y\right)$ acting along the *i*th line, either at the boundary or between two adjacent strips of the assembled plate, can be expanded into a sine Fourier series by Equation (8), whose coefficients $V_{im}$ and $M_{im}$ are:

(20) $$\begin{array}{cc} \; & V_{im}=\frac{2}{L}\int_{0}^{L}f_{i}\left(y\right)\sin\left(\alpha_{m}y\right)\;dy\\ \; & M_{im}=\frac{2}{L}\int_{0}^{L}m_{i}\left(y\right)\sin\left(\alpha_{m}y\right)\;dy \end{array}$$

**Figure 2:**
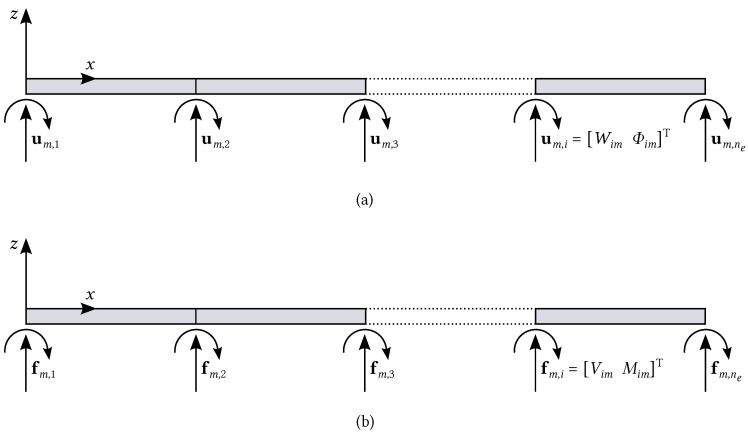
Plate consisting of $n_{e}-1$ strips: (**a**) displacements and (**b**) generalised forces.

The coefficients in Equation (20) are collected in the subvector $f_{m,i}={\left[\begin{array}{c} V_{im},M_{im} \end{array}\right]}^{T}$ of the force vector $f_{m}$ in Equation (6) (represented in Figure 2), which in turn is collected in the global force vector f in Equation (19). Once the vector f is built, the Fourier coefficients of the frequency response are given as:

(21) $$u={\tilde{D}}^{-1}\left(\omega \right)f$$

where $u={\left[\begin{array}{ccc} u_{1}^{T} & \ldots & u_{N}^{T} \end{array}\right]}^{T}$ collects displacement vectors associated with the terms of the Fourier series, i.e., $u_{m}={\left[\begin{array}{ccc} u_{m,1}^{T} & \ldots & u_{m,n_{e}}^{T} \end{array}\right]}^{T}$ with m=1,…,N and $u_{m,i}={\left[\begin{array}{cc} W_{im} & \Phi_{im} \end{array}\right]}^{T}$. The displacement $u_{i}\left(y\right)$ and rotation $\phi_{i}\left(y\right)$ along the ith line are simply given as:

(22) $$\begin{array}{cc} \; & u_{i}\left(y\right)=\sum_{m=1}^{\infty }W_{im}\sin\left(\alpha_{m}y\right)\\ \; & \phi_{i}\left(y\right)=\sum_{m=1}^{\infty }\Phi_{im}\sin\left(\alpha_{m}y\right) \end{array}$$

where $W_{im}$ and $\Phi_{im}$ are respectively the coefficients of the Fourier series expansion of the displacement and rotation along the *i*th line.

Notice that concentrated loads can readily be handled within the framework above: for this, two strips separated by a line passing through the load application point can be considered, and the load can be modelled as a standard 1D Dirac’s delta.

## 5. Numerical Applications

To validate the proposed method, consider a simply supported locally resonant steel plate coupled with 2-DOF viscously damped resonators and having dimensions 0.20m×0.20m×0.01m. The plate and resonator parameters are: Young’s modulus E=200GPa, Poisson’s ratio ν=0.3, volumetric mass density $\rho =7750\;\mathrm{kg}/m^{3}$, $k_{1}=k_{2}=10\;\mathrm{kN}/m$, $m_{1}=m_{2}=0.01\;\mathrm{kg}$, $c_{1}=c_{2}=0.05\;\mathrm{Ns}m^{-1}$.

The proposed dynamic-stiffness approach is implemented by applying the contour-integral algorithm to solve the eigenvalue problem involving the dynamic-stiffness matrix in Equation (18), where the frequency-dependent stiffness of the resonators is calculated as in [30]. The parameters of the contour-integral algorithm are $N_{0}=36$, $L_{0}=60$, K=15.

**Figure 3:**
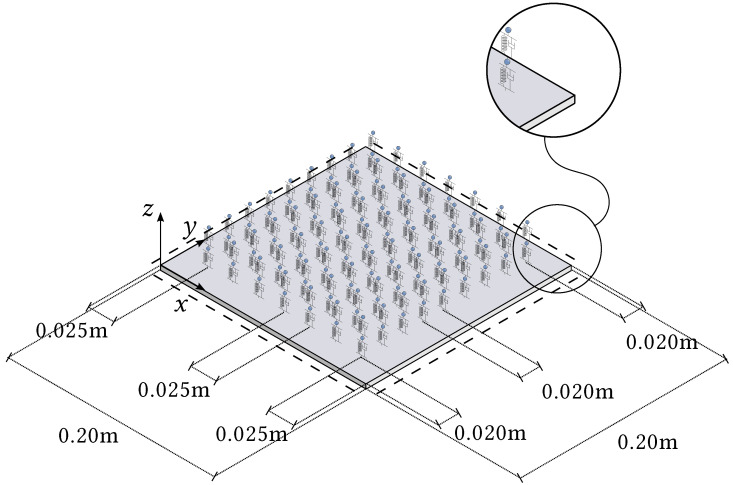
Simply supported LRMP with mass–spring–damper subsystems modelling 2-DOF resonators.

**Figure 4:**
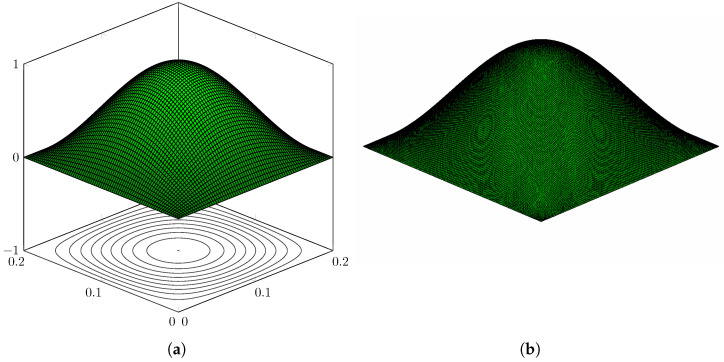
Simply supported LRMP in Figure 3, real part of Mode 127: (**a**) dynamic-stiffness approach and (**b**) finite-element method.

**Figure 5:**
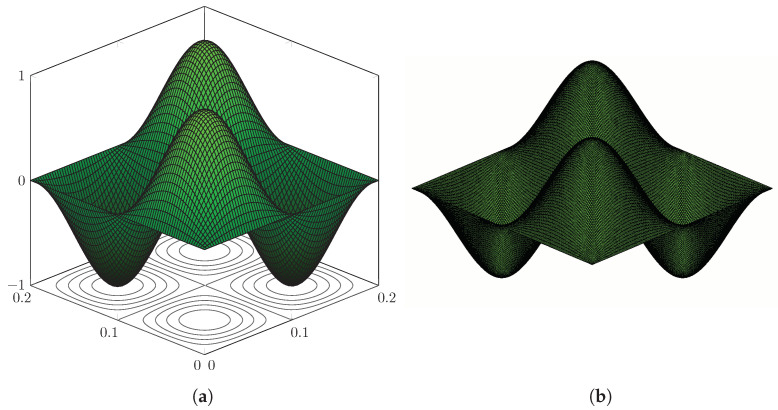
Simply supported LRMP in Figure 3, real part of Mode 130: (**a**) dynamic-stiffness approach and (**b**) finite-element method.

**Figure 6:**
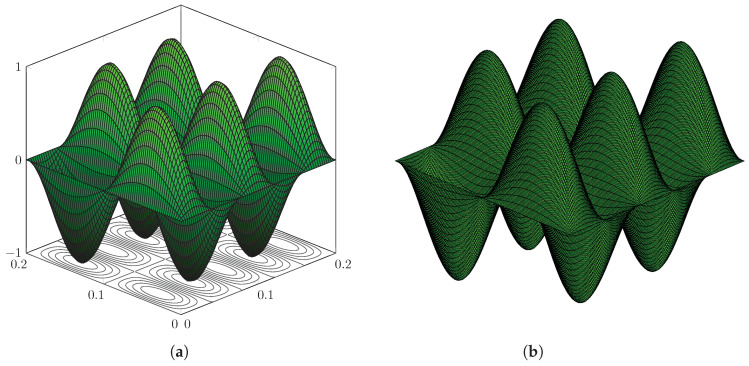
Simply supported LRMP in Figure 3, real part of Mode 144: (**a**) dynamic-stiffness approach and (**b**) finite-element method.

**Figure 7:**
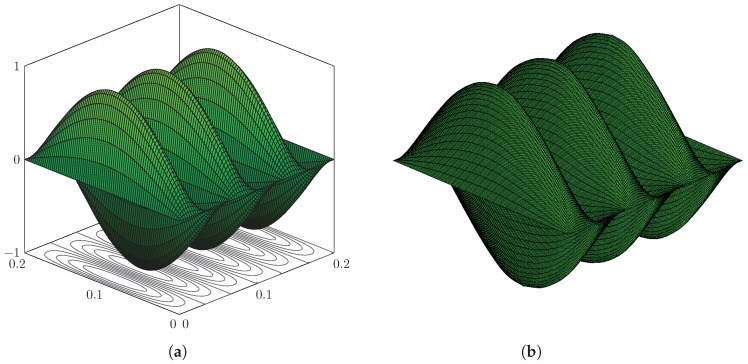
Simply supported LRMP in Figure 3, real part of Mode 150: (**a**) dynamic-stiffness approach and (**b**) finite-element method.

**Figure 8:**
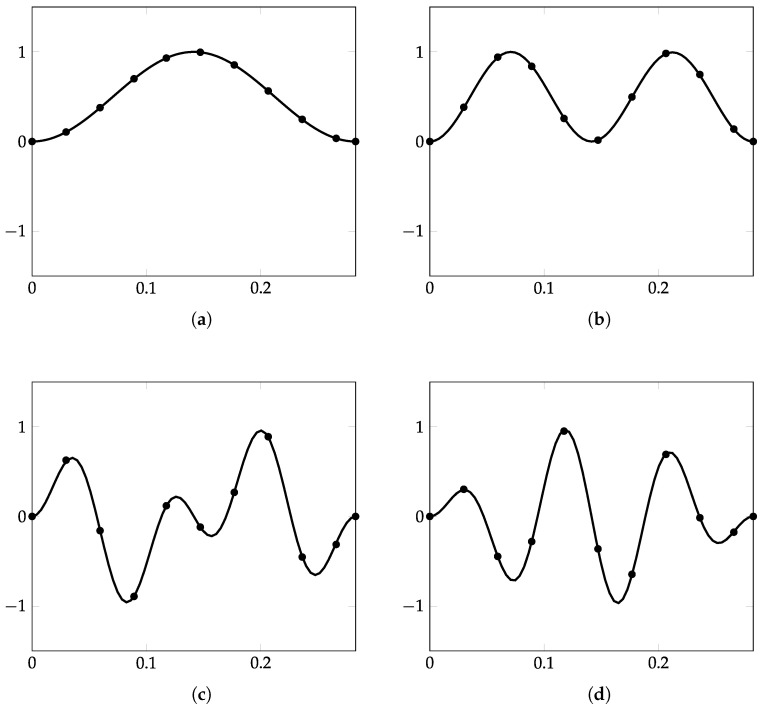
Simply supported LRMP in Figure 3, view along the line x=y of the real part of the modes: (**a**) 127, (**b**) 130, (**c**) 144, and (**d**) 150; dynamic-stiffness approach (black continuous line) and finite-element method in ABAQUS (black dots).

The first 150 eigenvalues are computed using the proposed method and, in order to assess the accuracy, modelling the plate in Figure 3 with the FE code Abaqus using a mesh of 200×200 S4R5 elements. Some eigenvalues are reported in Table 1, while the complete list of the first 150 is given in Table A1, Table A2, Table A3 in Appendix A, for conciseness. The calculated eigenvalues are in excellent agreement and the maximum relative error computed is equal to ϵ=3.65% for the real part and ϵ=0.78% for the imaginary part. Furthermore, it is seen that the contour-integral algorithm is capable of capturing eigenvalues very close to each other, which may differ even by a few digits. An excellent agreement is found in terms of mode shapes, as shown in Figure 4, Figure 5, Figure 6, Figure 7 and Figure 8. Notice that the mode shapes are complex, as they are associated with complex eigenvalues; see Table 1 and Table A1, Table A2, Table A3 in Appendix A; in particular, Figure 4, Figure 5, Figure 6, Figure 7 and Figure 8 show the dimensionless real parts, as obtained upon dividing all components by the one with the maximum absolute value.

As for the size of the two models, the proposed dynamic-stiffness model involves a 144×144 dynamic-stiffness matrix (corresponding to eight strips with nine generalised coordinates each), while the FE model involves 11,8791 × 11,8791 stiffness and mass matrices (corresponding to 40,401 nodes). It is seen that the eigenvalues and the maximum relative error do not change appreciably by increasing the sizes of the two models. Moreover, no significant differences are found in the eigenvalues if the parameters of the contour-integral algorithm are changed. As for the computational effort, it is noteworthy that the contour-integral algorithm is implemented in an in-house MATLAB code. For the parameters assumed, i.e., $N_{0}=36$, $L_{0}=60$, K=15, the computation time required to calculate the eigenvalues in Table 1 and Table A1, Table A2, Table A3 of Appendix A is 44.4 s. On the other hand, the corresponding eigenvalues of the finite-element model in ABAQUS are calculated by the default Lanczos eigensolver, the computation time of which is 111.2 s. Therefore, it can be concluded that the dynamic-stiffness model in conjunction with the contour-integral algorithm provides very accurate results compared to a standard FE approach, requiring a very limited size of the model and computational effort.

**Figure 9:**
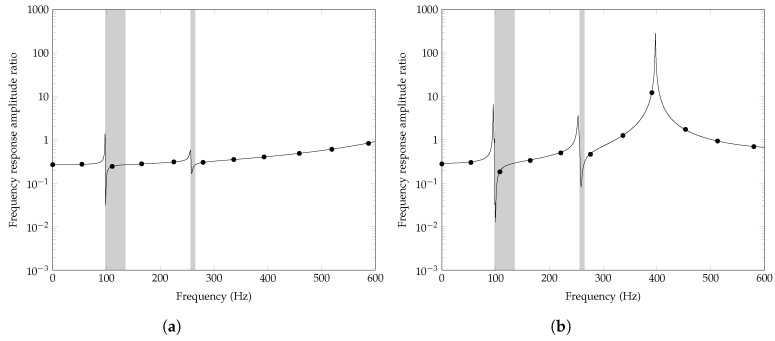
LRMP in Figure 3, amplitude ratio of the deflection frequency response at (0.175,0.10) to the deflection frequency response at the application point (0.025,0.10) of a unit harmonic force, calculated by the dynamic-stiffness approach (black continuous line) and FE model in ABAQUS (black dots), considering: (**a**) all edges simply supported; (**b**) two edges simply supported and two edges free.

Finally, the dynamic-stiffness approach proposed in Section 4 is applied to calculate the transmittance of the plate in Figure 3, considering two sets of BCs: (i) all edges are simply supported; (ii) two edges are simply supported and two are free. Specifically, a unit harmonic concentrated load is applied at $\left(x_{0},y_{0}\right)=\left(0.025,0.10\right)$, and the transmittance is evaluated as the ratio of the deflection at $\left(x_{1},y_{1}\right)=\left(0.175,0.10\right)$ to the deflection at the load application point $\left(x_{0},y_{0}\right)$. For completeness, Figure 9 includes the band gaps of the corresponding infinite LRMP, as computed by the standard FE approach [32]. The transmittance varies significantly with the BCs and, within the band gaps of the corresponding infinite plate, better wave attenuation properties of the finite plate are found when two edges of the plate are free and two simply supported, compared to the case where all the edges are simply supported.

## 6. Discussion

This paper has addressed the free vibrations of non-classically damped locally resonant metamaterial plates, focusing on Lévy plates. The main novelties of the proposed approach can be summarized as: (1) the formulation of a reduced-order dynamic-stiffness model where multi-degree-of-freedom resonators are represented via frequency-dependent reaction forces, involving the deflection of the attachment points only and no resonator DOFs; (2) the application of the contour-integral algorithm to calculate the complex eigenvalues; (3) a dynamic-stiffness approach to the calculation of the frequency response under arbitrarily placed concentrated loads, which is of interest to investigate the elastic wave attenuation properties of the plate. Considering a simply supported plate coupled with 2-DOF viscously damped resonators, it has been demonstrated that the proposed approach provides very accurate eigenvalues and mode shapes, with a very limited size of the model and computational costs compared to a standard FE approach implemented in Abaqus. It is noteworthy that the proposed approach requires only that the dynamic-stiffness matrix of the bare plate is available. As such, it is readily extendible to locally resonant metamaterial Kirchhoff plates with arbitrary BCs, using the appropriate dynamic-stiffness matrix formulated in [18,19,21].

It is important to remark that the LRMPs under study are of interest not only as structural/mechanical components at the macroscale, but also at much smaller scales. For instance, the concept of periodic arrays of resonators coupled with a primary host system was proposed at the microscale, e.g.,: Reference [33] investigated numerically and experimentally the formation of locally resonant band gaps in a 2D surface phononic crystal with inverted conical pillars; specifically, the inverted conical pillars were deposited on a semi-infinite lithium–niobate substrate and arranged in a honeycomb lattice array for applications in low-frequency guiding, acoustic wave isolation, acoustic absorbers, and acoustic filters. Moreover, numerical and experimental investigations demonstrated the existence of band gaps at a multi-GHz frequency range in a pillar-based hypersonic 2D phononic crystal with nanoscale dimensions [34]; in this case, the fabricated phononic crystal consisted of a periodic array of nanopillars arranged according to the triangular lattice structure of the crystal. An interesting discussion on the potential applications of locally resonant phononic nanostructures with other pertinent references may be found in the work by Guo et al. [35]. These recent studies demonstrate the interest in LRMPs at various scales, for which the proposed approach may represent a valuable analysis tool.

## Figures and Tables

**Table 1:** Complex eigenvalues (from 1 to 150) of the LRMP in Figure 3 with 2-DOF resonators, computed by means of the proposed dynamic-stiffness approach (DS) and the FE method (FE).

Mode	Eigenvalue ($\cdot 10^{4}$) (DS)	Eigenvalue ($\cdot 10^{4}$) (FE)
1	0.06099453±0.00009052i	0.06097390±0.00009040i
2	0.06167468±0.00009470i	0.06167134±0.00009468i
3	0.06167470±0.00009470i	0.06167133±0.00009468i
4	0.06175305±0.00009518i	0.06175079±0.00009517i
5	0.06177108±0.00009529i	0.06176983±0.00009528i
6	0.06177113±0.00009529i	0.06176980±0.00009528i
7	0.06178420±0.00009537i	0.06178295±0.00009537i
8	0.06178422±0.00009537i	0.06178293±0.00009537i
9	0.06179204±0.00009542i	0.06179124±0.00009542i
10	0.06179212±0.00009542i	0.06179117±0.00009542i
11	0.06179329±0.00009543i	0.06179227±0.00009542i
12	0.06179513±0.00009544i	0.06179431±0.00009544i
13	0.06179520±0.00009544i	0.06179425±0.00009544i
14	0.06179803±0.00009546i	0.06179724±0.00009545i
15	0.06179807±0.00009546i	0.06179761±0.00009546i
⋮	⋮	⋮
90	0.16179997±0.00065447i	0.16179932±0.00065446i
91	0.16180003±0.00065448i	0.16179955±0.00065447i
92	0.16180020±0.00065448i	0.16179805±0.00065444i
93	0.16180029±0.00065448i	0.16179969±0.00065447i
94	0.16180039±0.00065448i	0.16179984±0.00065447i
95	0.16180047±0.00065448i	0.16179996±0.00065447i
96	0.16180062±0.00065448i	0.16179999±0.00065447i
97	0.16180063±0.00065448i	0.16180003±0.00065448i
98	0.16180070±0.00065449i	0.16180008±0.00065448i
99	0.16180070±0.00065448i	0.16180010±0.00065448i
100	0.16180076±0.00065449i	0.16180016±0.00065448i
⋮	⋮	⋮
135	3.21092421±0.00012816i	3.15927753±0.00012834i
136	3.21092421±0.00012816i	3.15939426±0.00012844i
137	3.39884491±0.00012807i	3.31429443±0.00012829i
138	3.77441335±0.00012790i	3.68814316±0.00012808i
139	3.77441335±0.00012790i	3.68894501±0.00012809i
140	4.71172216±0.00012751i	4.57325706±0.00012775i
141	4.71172217±0.00012751i	4.57372169±0.00012786i
142	4.89890560±0.00012743i	4.79083290±0.00012797i
143	4.89890560±0.00012744i	4.79105072±0.00012793i
144	5.45989728±0.00012722i	5.30883064±0.00012762i
145	5.45989728±0.00012722i	5.30934525±0.00012751i
146	6.02005043±0.00012701i	5.80780907±0.00012723i
147	6.39302010±0.00012688i	6.17382736±0.00012755i
148	6.39302011±0.00012688i	6.17507515±0.00012727i
149	6.95177672±0.00012667i	6.74670029±0.00012756i
150	6.95177673±0.00012668i	6.74753723±0.00012768i

## Data Availability

The raw/processed data required to reproduce the above findings cannot be shared at this time as the data also forms part of an ongoing study.

## Abbreviations

The following abbreviations are used in this manuscript:

BC	Boundary condition
DOF	Degree of freedom
FE	Finite element
LRMP	Locally resonant metamaterial plate

## Appendix A. Eigenvalues

**Table A1:** Complex eigenvalues (from 1 to 50) of the LRMP in Figure 3 with 2-DOF resonators, computed by means of the proposed dynamic-stiffness approach (DS) and the FE method (FE).

Mode	Eigenvalue ($\cdot 10^{4}$) (DS)	Eigenvalue ($\cdot 10^{4}$) (FE)
1	0.06099453±0.00009052i	0.06097390±0.00009040i
2	0.06167468±0.00009470i	0.06167134±0.00009468i
3	0.06167470±0.00009470i	0.06167133±0.00009468i
4	0.06175305±0.00009518i	0.06175079±0.00009517i
5	0.06177108±0.00009529i	0.06176983±0.00009528i
6	0.06177113±0.00009529i	0.06176980±0.00009528i
7	0.06178420±0.00009537i	0.06178295±0.00009537i
8	0.06178422±0.00009537i	0.06178293±0.00009537i
9	0.06179204±0.00009542i	0.06179124±0.00009542i
10	0.06179212±0.00009542i	0.06179117±0.00009542i
11	0.06179329±0.00009543i	0.06179227±0.00009542i
12	0.06179513±0.00009544i	0.06179431±0.00009544i
13	0.06179520±0.00009544i	0.06179425±0.00009544i
14	0.06179803±0.00009546i	0.06179724±0.00009545i
15	0.06179807±0.00009546i	0.06179761±0.00009546i
16	0.06179834±0.00009546i	0.06179774±0.00009546i
17	0.06179849±0.00009546i	0.06179721±0.00009545i
18	0.06179928±0.00009547i	0.06179866±0.00009546i
19	0.06179941±0.00009547i	0.06179855±0.00009546i
20	0.06180004±0.00009547i	0.06179963±0.00009547i
21	0.06180033±0.00009547i	0.06179971±0.00009547i
22	0.06180043±0.00009547i	0.06179929±0.00009547i
23	0.06180064±0.00009547i	0.06180020±0.00009547i
24	0.06180090±0.00009548i	0.06180034±0.00009547i
25	0.06180100±0.00009548i	0.06180053±0.00009547i
26	0.06180122±0.00009548i	0.06180054±0.00009547i
27	0.06180123±0.00009548i	0.06180059±0.00009547i
28	0.06180128±0.00009548i	0.06179999±0.00009547i
29	0.06180145±0.00009548i	0.06180092±0.00009548i
30	0.06180154±0.00009548i	0.06180095±0.00009548i
31	0.06180164±0.00009548i	0.06180109±0.00009548i
32	0.06180172±0.00009548i	0.06180121±0.00009548i
33	0.06180187±0.00009548i	0.06180123±0.00009548i
34	0.06180188±0.00009548i	0.06180127±0.00009548i
35	0.06180195±0.00009548i	0.06180133±0.00009548i
36	0.06180196±0.00009549i	0.06180134±0.00009548i
37	0.06180201±0.00009548i	0.06180141±0.00009548i
38	0.06180210±0.00009548i	0.06180079±0.00009548i
39	0.06180220±0.00009548i	0.06180159±0.00009548i
40	0.06180223±0.00009549i	0.06180160±0.00009548i
41	0.06180227±0.00009549i	0.06180166±0.00009548i
42	0.06180231±0.00009549i	0.06180178±0.00009548i
43	0.06180240±0.00009549i	0.06180179±0.00009548i
44	0.06180245±0.00009549i	0.06180181±0.00009548i
45	0.06180246±0.00009549i	0.06180184±0.00009548i
46	0.06180251±0.00009549i	0.06180187±0.00009548i
47	0.06180254±0.00009549i	0.06180193±0.00009548i
48	0.06180261±0.00009549i	0.06180195±0.00009548i
49	0.06180263±0.00009549i	0.06180200±0.00009548i
50	0.06180264±0.00009549i	0.06180201±0.00009548i

**Table A2:** Complex eigenvalues (from 51 to 100) of the LRMP in Figure 3 with 2-DOF resonators, computed by means of the proposed dynamic-stiffness approach (DS) and the FE method (FE).

Mode	Eigenvalue ($\cdot 10^{4}$) (DS)	Eigenvalue ($\cdot 10^{4}$) (FE)
51	0.06180269±0.00009550i	0.06180204±0.00009548i
52	0.06180273±0.00009549i	0.06180205±0.00009548i
53	0.06180277±0.00009549i	0.06180207±0.00009548i
54	0.06180278±0.00009551i	0.06180211±0.00009548i
55	0.06180279±0.00009549i	0.06180213±0.00009548i
56	0.06180280±0.00009549i	0.06180214±0.00009548i
57	0.06180281±0.00009548i	0.06180217±0.00009548i
58	0.06180287±0.00009549i	0.06180220±0.00009548i
59	0.06180289±0.00009553i	0.06180158±0.00009548i
60	0.06180290±0.00009549i	0.06180224±0.00009548i
61	0.06180290±0.00009546i	0.06180227±0.00009548i
62	0.06180294±0.00009549i	0.06180230±0.00009549i
63	0.06180297±0.00009548i	0.06180222±0.00009548i
64	0.16086702±0.00063822i	0.16083957±0.00063771i
65	0.16167059±0.00065236i	0.16166713±0.00065230i
66	0.16167061±0.00065236i	0.16166711±0.00065230i
67	0.16175137±0.00065368i	0.16174909±0.00065365i
68	0.16176966±0.00065398i	0.16176840±0.00065396i
69	0.16176970±0.00065398i	0.16176838±0.00065396i
70	0.16178289±0.00065420i	0.16178165±0.00065418i
71	0.16178291±0.00065420i	0.16178162±0.00065418i
72	0.16179077±0.00065433i	0.16178997±0.00065431i
73	0.16179085±0.00065433i	0.16179100±0.00065433i
74	0.16179202±0.00065435i	0.16178991±0.00065431i
75	0.16179387±0.00065438i	0.16179304±0.00065436i
76	0.16179394±0.00065438i	0.16179299±0.00065436i
77	0.16179677±0.00065442i	0.16179599±0.00065441i
78	0.16179681±0.00065442i	0.16179637±0.00065442i
79	0.16179708±0.00065443i	0.16179649±0.00065442i
80	0.16179723±0.00065443i	0.16179730±0.00065443i
81	0.16179803±0.00065444i	0.16179742±0.00065443i
82	0.16179816±0.00065445i	0.16179596±0.00065441i
83	0.16179879±0.00065446i	0.16179838±0.00065445i
84	0.16179908±0.00065446i	0.16179845±0.00065445i
85	0.16179918±0.00065446i	0.16179873±0.00065445i
86	0.16179939±0.00065447i	0.16179895±0.00065446i
87	0.16179965±0.00065447i	0.16179910±0.00065446i
88	0.16179975±0.00065447i	0.16179928±0.00065446i
89	0.16179997±0.00065447i	0.16179929±0.00065446i
90	0.16179997±0.00065447i	0.16179932±0.00065446i
91	0.16180003±0.00065448i	0.16179955±0.00065447i
92	0.16180020±0.00065448i	0.16179805±0.00065444i
93	0.16180029±0.00065448i	0.16179969±0.00065447i
94	0.16180039±0.00065448i	0.16179984±0.00065447i
95	0.16180047±0.00065448i	0.16179996±0.00065447i
96	0.16180062±0.00065448i	0.16179999±0.00065447i
97	0.16180063±0.00065448i	0.16180003±0.00065448i
98	0.16180070±0.00065449i	0.16180008±0.00065448i
99	0.16180070±0.00065448i	0.16180010±0.00065448i
100	0.16180076±0.00065449i	0.16180016±0.00065448i

**Table A3:** Complex eigenvalues (from 101 to 150) of the LRMP in Figure 3 with 2-DOF resonators, computed by means of the proposed dynamic-stiffness approach (DS) and the FE method (FE).

Mode	Eigenvalue ($\cdot 10^{4}$) (DS)	Eigenvalue ($\cdot 10^{4}$) (FE)
101	0.16180085±0.00065449i	0.16180033±0.00065448i
102	0.16180095±0.00065449i	0.16180034±0.00065448i
103	0.16180098±0.00065449i	0.16180035±0.00065448i
104	0.16180103±0.00065449i	0.16180041±0.00065448i
105	0.16180106±0.00065449i	0.16180053±0.00065448i
106	0.16180119±0.00065449i	0.16180054±0.00065448i
107	0.16180121±0.00065449i	0.16180056±0.00065448i
108	0.16180125±0.00065450i	0.16180060±0.00065448i
109	0.16180126±0.00065450i	0.16180062±0.00065449i
110	0.16180129±0.00065449i	0.16180068±0.00065449i
111	0.16180136±0.00065450i	0.16180069±0.00065449i
112	0.16180136±0.00065450i	0.16180074±0.00065449i
113	0.16180139±0.00065450i	0.16180075±0.00065449i
114	0.16180146±0.00065450i	0.16180078±0.00065449i
115	0.16180148±0.00065450i	0.16180079±0.00065449i
116	0.16180152±0.00065450i	0.16180081±0.00065449i
117	0.16180153±0.00065450i	0.16180085±0.00065449i
118	0.16180155±0.00065450i	0.16180087±0.00065449i
119	0.16180156±0.00065450i	0.16180089±0.00065449i
120	0.16180161±0.00065450i	0.16180092±0.00065449i
121	0.16180162±0.00065450i	0.16180096±0.00065449i
122	0.16180164±0.00065450i	0.16180098±0.00065449i
123	0.16180165±0.00065450i	0.16180099±0.00065449i
124	0.16180169±0.00065450i	0.16180102±0.00065449i
125	0.16180172±0.00065450i	0.16180105±0.00065449i
126	0.16180228±0.00065458i	0.16179967±0.00065447i
127	0.38637283±0.00015015i	0.38188243±0.00015078i
128	0.94979965±0.00013164i	0.93953762±0.00013174i
129	0.94979965±0.00013164i	0.93953552±0.00013173i
130	1.51582776±0.00012963i	1.48995104±0.00012971i
131	1.89304723±0.00012909i	1.87023849±0.00012922i
132	1.89304723±0.00012909i	1.87044411±0.00012920i
133	2.45835312±0.00012860i	2.41032791±0.00012871i
134	2.45835312±0.00012860i	2.41037235±0.00012874i
135	3.21092421±0.00012816i	3.15927753±0.00012834i
136	3.21092421±0.00012816i	3.15939426±0.00012844i
137	3.39884491±0.00012807i	3.31429443±0.00012829i
138	3.77441335±0.00012790i	3.68814316±0.00012808i
139	3.77441335±0.00012790i	3.68894501±0.00012809i
140	4.71172216±0.00012751i	4.57325706±0.00012775i
141	4.71172217±0.00012751i	4.57372169±0.00012786i
142	4.89890560±0.00012743i	4.79083290±0.00012797i
143	4.89890560±0.00012744i	4.79105072±0.00012793i
144	5.45989728±0.00012722i	5.30883064±0.00012762i
145	5.45989728±0.00012722i	5.30934525±0.00012751i
146	6.02005043±0.00012701i	5.80780907±0.00012723i
147	6.39302010±0.00012688i	6.17382736±0.00012755i
148	6.39302011±0.00012688i	6.17507515±0.00012727i
149	6.95177672±0.00012667i	6.74670029±0.00012756i
150	6.95177673±0.00012668i	6.74753723±0.00012768i
